# Lyophilized Platelet-Rich Fibrin (PRF) Promotes Craniofacial Bone Regeneration through Runx2

**DOI:** 10.3390/ijms15058509

**Published:** 2014-05-14

**Authors:** Qi Li, David A. Reed, Liu Min, Gokul Gopinathan, Steve Li, Smit J. Dangaria, Leo Li, Yajun Geng, Maria-Therese Galang, Praveen Gajendrareddy, Yanmin Zhou, Xianghong Luan, Thomas G. H. Diekwisch

**Affiliations:** 1 Department of Implantology, Stomatological Hospital, Jilin University, Changchun 130021, Jilin, China; E-Mails: gyjlq@163.com (Q.L.); liqigyj@gmail.com (Y.G.); zhouym@jlu.edu.cn (Y.Z.); 2 UIC Brodie Laboratory for Craniofacial Genetics, Chicago, IL 60612, USA; E-Mails: reedd@uic.edu (D.A.R.); ggokul@uic.edu (G.G.); shitianli@gmail.com (S.L.); leonardo3445@gmail.com (L.L.); luan@uic.edu (X.L.); 3 Department of Periodontology, Stomatological Hospital, Jilin University, Changchun 130021, Jilin, China; E-Mail: liu-min99@jlu.edu.cn; 4 College of Dentistry, University of Southern California, Los Angeles, CA 90089, USA; E-Mail: dangaria@gmail.com; 5 Department of Orthodontics, College of Dentistry, University of Illinois at Chicago, Chicago, IL 60612, USA; E-Mail: mgalang@uic.edu; 6 Department of Periodontics, College of Dentistry, University of Illinois at Chicago, Chicago, IL 60612, USA; E-Mail: praveen@uic.edu

**Keywords:** Platelet-rich fibrin, lyophilization, calvaria, alveolar bone, Runx2

## Abstract

Freeze-drying is an effective means to control scaffold pore size and preserve its composition. The purpose of the present study was to determine the applicability of lyophilized Platelet-rich fibrin (LPRF) as a scaffold for craniofacial tissue regeneration and to compare its biological effects with commonly used fresh Platelet-rich fibrin (PRF). LPRF caused a 4.8-fold ± 0.4-fold elevation in Runt-related transcription factor 2 (Runx2) expression in alveolar bone cells, compared to a 3.6-fold ± 0.2-fold increase when using fresh PRF, and a more than 10-fold rise of alkaline phosphatase levels and mineralization markers. LPRF-induced Runx2 expression only occurred in alveolar bone and not in periodontal or dental follicle cells. LPRF also caused a 1.6-fold increase in osteoblast proliferation (*p* < 0.001) when compared to fresh PRF. When applied in a rat craniofacial defect model for six weeks, LPRF resulted in 97% bony coverage of the defect, compared to 84% for fresh PRF, 64% for fibrin, and 16% without scaffold. Moreover, LPRF thickened the trabecular diameter by 25% when compared to fresh PRF and fibrin, and only LPRF and fresh PRF resulted in the formation of interconnected trabeculae across the defect. Together, these studies support the application of lyophilized PRF as a biomimetic scaffold for craniofacial bone regeneration and mineralized tissue engineering.

## 1. Introduction

Platelet-rich fibrin (PRF) is a second generation platelet concentrate developed as an improvement over the earlier introduced platelet-rich plasma (PRP) as an aid for tissue repair and regeneration [1]. In contrast to PRP, which is prepared by adding bovine thrombin and anticoagulants, PRF is prepared from centrifuged blood and is strictly autologous. PRF predominantly consists of a fibrin matrix rich in platelet and leukocyte cytokines such as IL-1, -4, -6, and growth factors such as Transforming Growth Factor beta 1 (TGF-β1), Platelet Derived Growth Factor (PDGF), and Vascular Endothelial Growth Factor (VEGF). Fibrin gels exploit the final stage of the coagulation cascade in which fibrinogen molecules self-assemble into a highly biocompatible three-dimensional fiber network [2]. The combination of fibrins and cytokines within PRF becomes a powerful bioscaffold with an integrated reservoir of growth factors for tissue regeneration [3]. The suitability of PRF as a biologically active scaffold has been illustrated in a number of studies revealing proliferation and differentiation of osteoblasts and gingival fibroblasts [4,5]. Clinical studies have demonstrated that PRF is applicable for soft tissue and bone regeneration [6,7], plastic surgery [8], periodontal tissue regeneration [9,10], and tendon repair [11,12]. The ability of PRF to augment and regenerate compromised tissues may be enhanced in combination with bone substitutes such as Bio-Oss^®^ or autologous bone [13,14]. Together, these studies have established PRF as a highly biocompatible and inductive bioscaffold useful for a broad range of tissue engineering applications.

As a fresh plasma preparation, PRF has been originally developed for immediate autologous use as a healing or regenerative scaffold, and reports in the literature emphasize same-day application to maximize the release of growth factors [1,5]. Therefore, the current benefits of PRF as a bioactive scaffold are limited to short-term application. Storage and/or international transfer using liquid nitrogen and dry ice pose safety and logistic concerns [15,16]. To address the issue of PRF storage and delayed clinical application, we have here focused on the development of a protocol for the use of lyophilized PRF. Freeze drying (lyophilization) is a commonly used process to improve the stability and long-term storage of proteins used for tissue regeneration [17,18]. Freeze-dried, protein-based materials not only have the advantage of better stability and storage potential, but also provide newly growing cells and tissues with immediate access to growth factors [19].

In the present study, we have examined the use of lyophilized PRF as a scaffold material for bone regeneration. Earlier studies have reported that PRF modulates cell proliferation and upregulates alkaline phosphatase activity of periodontal ligament cells [10,20]. Here, we have compared the effects of fresh and lyophilized PRF on dental follicle, periodontal ligament, and alveolar bone cells using biological assays for migration, proliferation, mineralization, and gene expression. The suitability of these cells for tissue regeneration applications have been reported in earlier studies [21–23]. We also tested PRF scaffold properties in subcutaneous implants in nude rats. Together, these studies for the first time examine and report the use of lyophilized PRF as a novel bioactive scaffold for tissue engineering applications.

## 2. Results

### 2.1. Lyophilized Platelet-Rich Fibrin (PRF) Featured a Sponge-Like Microstructure with 13.4-Fold Larger Pores Compared to Fresh PRF

**Figure 1.**
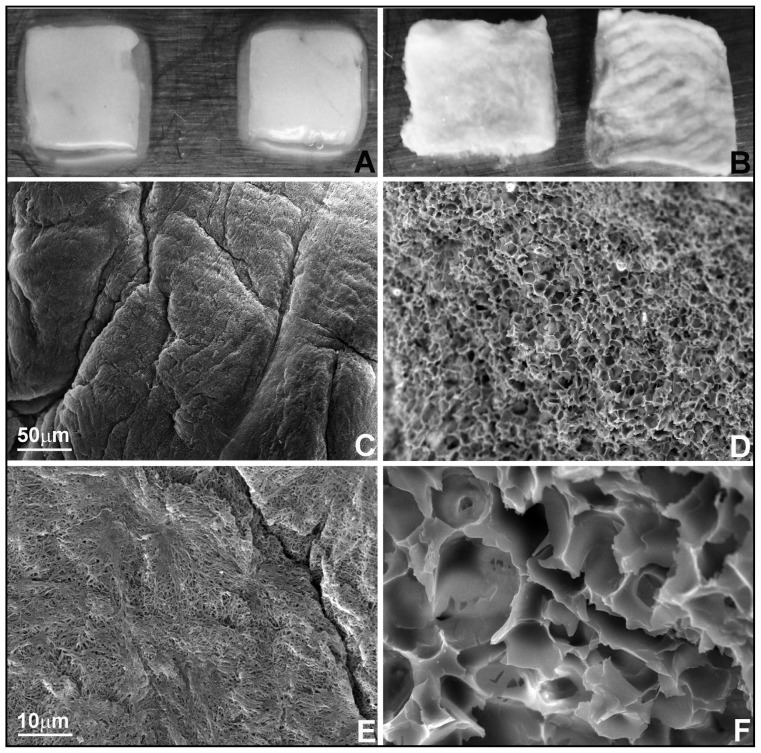
Macro-, micro-, and submicrographic comparison of fresh and lyophilized PRF structure. (**A**,**B**) are macrophotographs of fresh and lyophilized Platelet-rich fibrin (PRF) preparations, showing cell-free preparations on the left, while the preparations on the right contained red blood cells; (**C**–**F**) are scanning electron micrographs at 300-fold magnification (**C**,**D**) and 2000-fold magnification (**E**,**F**). (**A**,**C**,**E**) are from fresh PRF and (**B**,**D**,**F**) are from lyophilized PRF. A scale bar in the left-hand panel serves as a reference for the level of magnification in both panels.

The focus of the present study was to determine the biological properties and suitability of fresh and lyophilized PRF for tissue engineering. As a first step, PRF was prepared from fresh blood and either left untreated (Figure 1A) or lyophilized (Figure 1B). Surface parameters such as topography and hardness greatly affect cell behavior and gene expression [23]. To compare surface topography of fresh and lyophilized PRF, freshly cut samples were subjected to scanning electron microscopy (Figure 1C–F). Image analysis revealed that fresh PRF featured a fiber-like appearance with 0.6 ± 0.13 μm diameter pores (Figure 1E), while lyophilized PRF resembled a sponge containing 8.06 ± 0.31 μm diameter pores, resulting in a 13.4-fold larger pore size in lyophilized *versus* fresh PRF (Figure 1F *versus* Figure 1E).

### 2.2. Lyophilized PRF Improved Cell Proliferation and Migration of Periodontal Progenitors in Vitro when Compared to Fresh PRF and DMEM Medium

**Figure 2.**
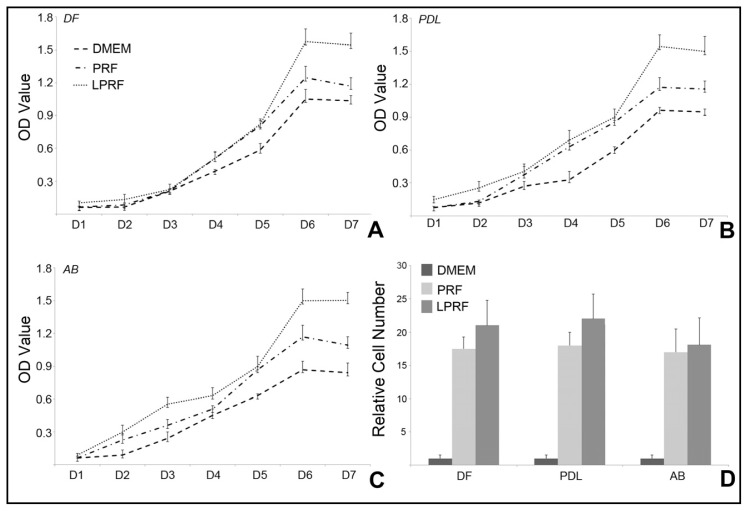
Effects of fresh and lyophilized PRF on proliferation and migration of periodontal progenitor populations. (**A**–**C**) illustrate the results of 3-(4,5-dimethylthiazol-2-yl)-2,5-diphenyltetrazolium bromide colorimetric proliferation assays when dental follicle progenitors (**A**), periodontal ligament progenitors (**B**), and alveolar bone osteoblast progenitors (**C**) were cultured on PRF-related substrates. The three different substrates used in the proliferation study, fresh PRF, lyophilized PRF, and DMEM medium, are distinguished by line patterns (**A**); (**D**) Difference in chemotaxis behavior between periodontal progenitors when cultured in PRF-conditioned media and DMEM medium. The three experimental groups are distinguished by shades of gray.

Application of lyophilized scaffolds for tissue engineering purposes involves consecutive events of freeze-drying and rehydration, which may affect membranes, structures, and factors residing within the scaffold [24,25]. To determine the effect of freeze-drying and subsequent rehydration as it relates to the use of PRF in tissue engineering, we performed a serious of studies on cellular functions of periodontal progenitors treated with fresh or lyophilized PRF. To determine the ability of PRF to promote cell proliferation and migration, periodontal progenitors were cultured for up to seven days using either fresh or lyophilized PRF compared DMEM alone (Figure 2A–C). Our cell proliferation assays using periodontal ligament fibroblasts (PDL), dental follicle progenitors (DF), and alveolar bone osteoblasts (AB), demonstrated a gradual increase in cell density over a culture period of seven days with all three culture conditions (Figure 2A–C). Both fresh and lyophilized PRF conditional media resulted in higher proliferation rates than DMEM medium (Figure 2A–C). After six days of culture, the lyophilized PRF induced increase in proliferation surpassed the fresh-PRF proliferation rate by 30%–40% in all cell cultures examined (*p* < 0.01) (Figure 2A–C). Migration assays revealed that both lyophilized and fresh PRF caused a highly significant (*p* < 0.0001) 15- to 20-fold increase in the number of migrated cells when compared to DMEM medium (Figure 2D). Lyophilized PRF slightly increased the number of migrated cells (*p* < 0.05) when compared to fresh PRF (Figure 2D).

### 2.3. Lyophilized PRF Enhanced the Mineralization Activity of Periodontal Progenitors in Vitro

**Figure 3.**
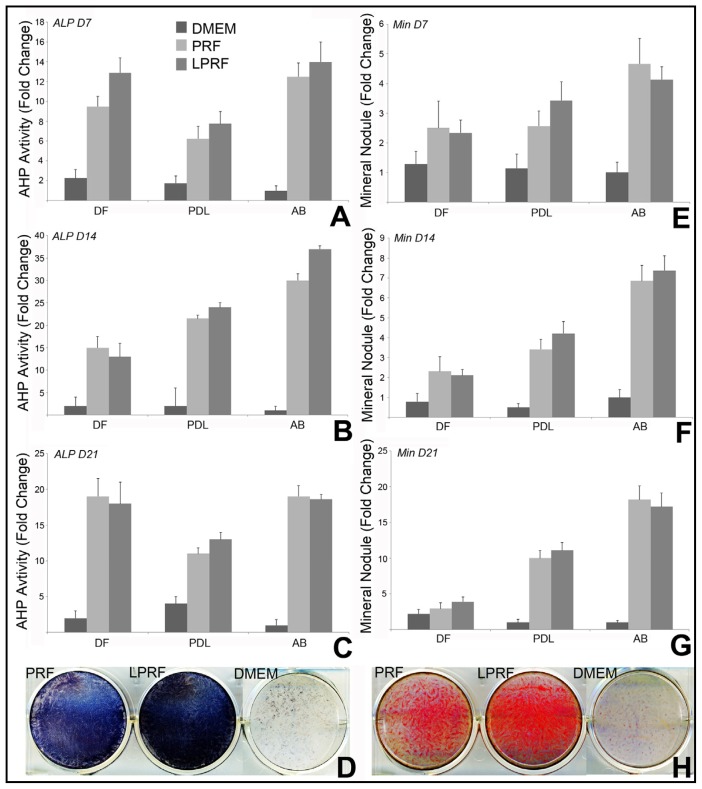
Effect of fresh and lyophilized PRF, and DMEM medium on mineralization behavior of periodontal progenitor populations. (**A**–**D**) are based on results from alkaline phosphatase staining assays and (**E**–**H**) are based on alizarin red S mineralization assays. In (**A**–**C**,**E**–**G**), alkaline phosphatase or alizarin red staining in periodontal progenitor cells cultured for 7, 14, and 21 days were compared. Different co-culture conditions (fresh or lyophilized PRF and DMEM) are distinguished by different bar patterns which are identified in the bar legend above Figure A. The three periodontal progenitor populations compared in this study, dental follicle, periodontal ligament, and alveolar bone are labeled on the *x*-axis of the graphs in (**A**–**C**,**E**–**G**); (**D**,**H**) illustrate the differences in mineralization behavior between alveolar bone progenitors co-cultured with fresh or lyophilized PRF and DMEM; (**D**) is a photograph of the alkaline phosphate stained 6-well plate and (**H**) is a photograph of the alizarin red stained 6-well plate.

To determine the effect of lyophilized PRF on the differentiation potential of periodontal progenitors, dental follicle, periodontal progenitors and alveolar bone osteoblasts were exposed to osteogenic induction condition in fresh or lyophilized PRF conditional medium or DMEM medium for 7, 14, and 21 days. Both fresh and lyophilized PRF demonstrated osteoinductive properties and significantly increased alkaline phosphatase activity (Figure 3A–D) and mineral nodule formation (Figure 3E–H) when compared to DMEM control. Lyophilized PRF strongly enhanced the osteogenic capacity of PDL and AB cells, showing higher ALP activity and increased mineral nodule formation at all three time points of the experiments (Figure 3A–H). However, PRF and LPRF had less of an osteoinductive effect in DF cells (Figure 3E–H).

### 2.4. Lyophilized PRF Upregulated RunX2 Expression and Modulated Matrix Gla Protein Gene Expression in Alveolar Bone Cells

**Figure 4.**
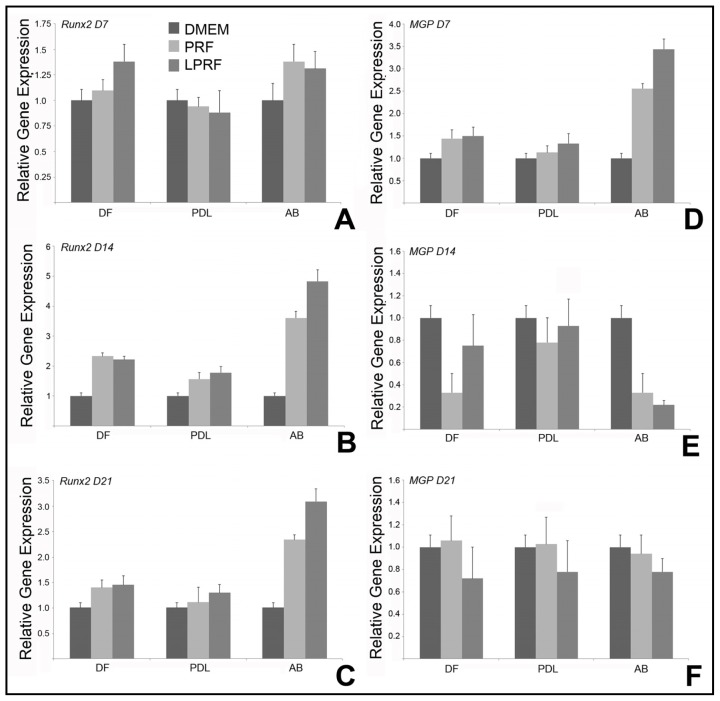
Differences in mineralization-associated gene expression patterns in co-cultures of periodontal progenitors and centrifuged blood derivatives. (**A**–**C**) are real-time RT-PCR assays for the osteoblast transcription factor Runx2; and (**D**–**F**) are real-time RT-PCR assays for the calcification inhibitor Matrix Gla Protein. Different co-culture conditions (fresh or lyophilized PRF and DMEM) are distinguished by different bar patterns identified in the upper right corner of the figure. The three periodontal progenitor populations compared in this study, dental follicle, periodontal ligament, and alveolar bone are labeled on the *x*-axis of the graphs in (**A**–**F**).

To determine molecular mechanisms by which PRFs modulate mineralization behavior of periodontal progenitor cells, quantitative real time RT-PCR was performed based on mRNA from DF, PDL and AB cells, which were treated with fresh or lyophilized PRF conditional media, or DMEM medium under osteoinductive conditions. In alveolar bone cells, expression of the osteoblast differentiation factor Runx2 increased on day 7, peaked on day 14 and remained upregulated on day 21 in all three cell types studied when comparing fresh and lyophilized PRF to DMEM (Figure 4A–C). There was no significant difference in Runx2 gene expression between fresh and lyophilized PRF in DF and PDL cells (Figure 4A–C). However, the effect of lyophilized PRF on RunX2 gene expression was significant in AB cells, revealing a 1.3-fold ± 0.2-fold elevation on day 14 and a 1.4-fold ± 0.1-fold increase on day 21 when compared to fresh PRF (*p* < 0.001) (Figure 4A–C). In contrast, both PRF and LPRF caused a significant 2.6-fold ± 0.1-fold (PRF)/3.4-fold ± 0.2-fold (LPRF) upregulation of the mineralization inhibitor matrix gla protein in alveolar bone progenitors on day 7, followed by a significant three-fold (PRF) or 4.5-fold (LPRF) downregulation on day 14 (*p* < 0.001) (Figure 4D–F), and the effect of LPRF was stronger than the effect of PRF on both days. After 21 days, there was little difference in MGP expression in all three cell types between DMEM, PRF, and LPRF. Remarkably, the significant effects of both PRF and LPRF on mineralization genes were limited to alveolar bone cells and were not detected in the other two cell types studied, namely PDL and DF progenitors.

### 2.5. Lyophilized PRF Promoted Cell Homing and Collagen Synthesis in Subcutaneous Implants

**Figure 5.**
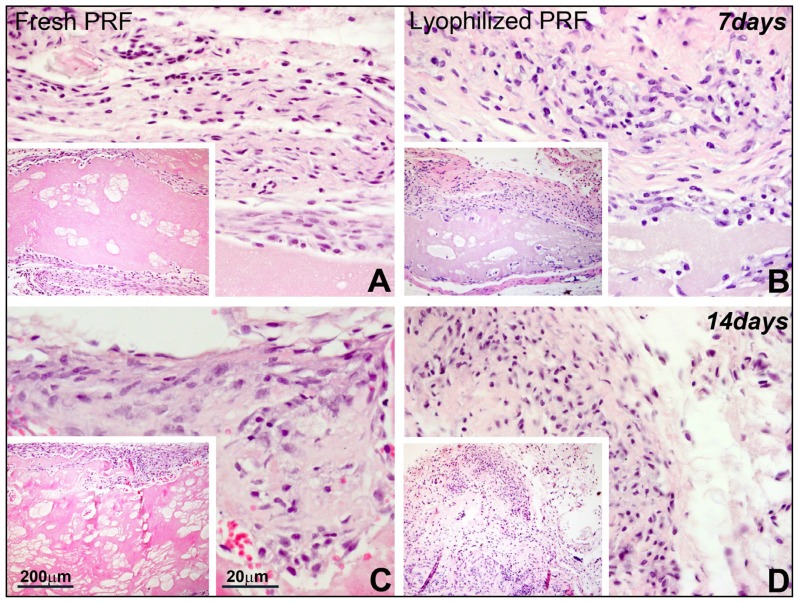
Comparison between lyophilized and fresh PRF in subcutaneous implants. Subcutaneous implants of fresh (**A**,**C**) and lyophilized PRF (**B**,**D**) were dissected from nude mice after seven days (**A**,**B**) and 14 days (**C**,**D**) of implantation. The samples were fixed and processed for paraffin sections and stained with hematoxylin and eosin. Note the loose and porous structure of the lyophilized PRF (**B**) when compared to fresh PRF (**A**) after seven days of implantation. The lyophilized PRF scaffold was infiltrated by fibroblast-like cells (**D**), while the fresh PRF remained intact (**C**) after 14 days of implantation.

To examine the *in vivo* applicability of PRFs as scaffold for stem cell recruitment into implanted tissue scaffolds, either fresh or lyophilized PRF, or fibrin gel were implanted subcutaneously into nude rats. In these subcutaneous implants, fresh PRF largely remained intact and separated from adjacent tissues (Figure 5A,C), while lyophilized PRF was penetrated by surrounding cells, resulting in improved tissue integration (Figure 5B–D). There was noticeable synthesis of thick collagen fibers identified by methyl blue staining in the lyophilized PRF scaffold, and collagen fibers were thicker in lyophilized PRF compared to those in fresh PRF and fibrin gel (not shown).

### 2.6. Lyophilized PRF Enhanced Cranial Bone Regeneration

**Figure 6.**
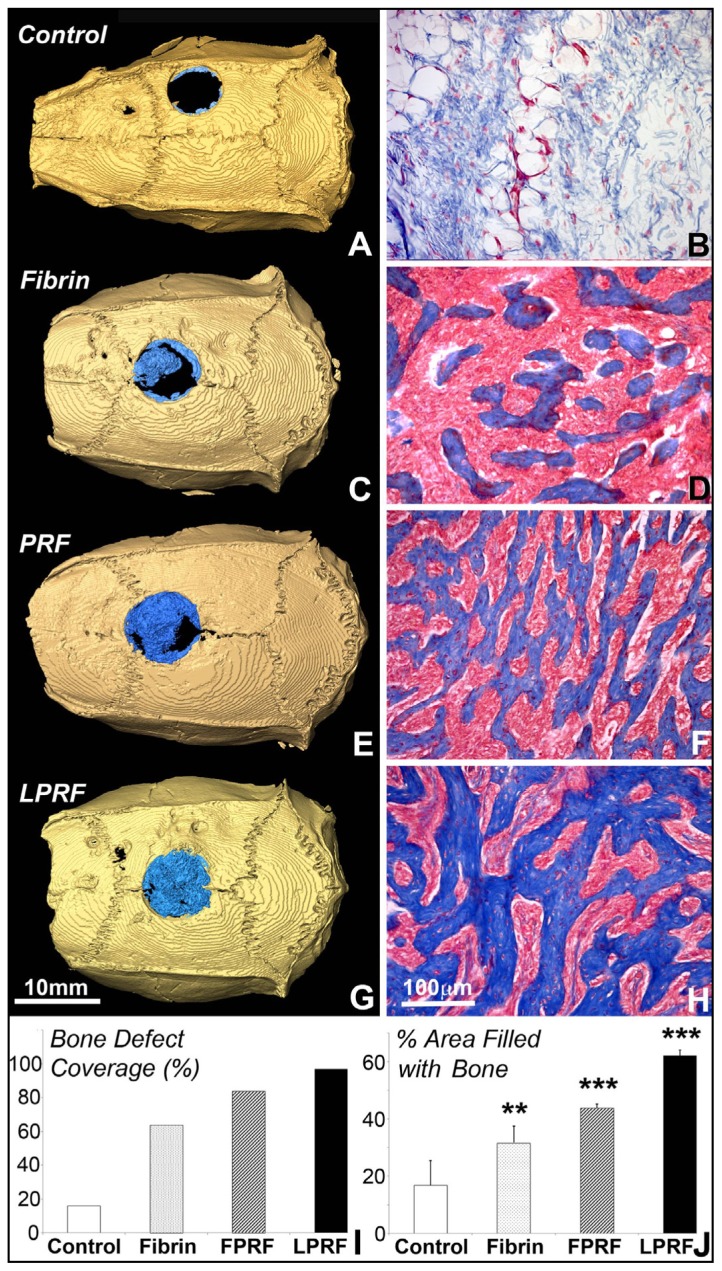
Analysis of bone regeneration in critical size calvarial defects using fibrin gel, fresh and lyophilized PRF as scaffolds. (**A**,**C**,**E**,**G**) are three-dimensional cranial microCT images of control (**A**), fibrin gel (**C**), fresh PRF (**E**) and lyophilized PRF (**G**) after six weeks of implantation. Regenerated bone was visualized in blue; (**B**,**D**,**F**,**H**) are photomicrographs of decalcified paraffin sections through control (**B**), fibrin gel (**D**), fresh PRF (**F**), and lyophilized PRF (**H**) six-week implants stained with Mallory’s procedure. Collagen-containing trabecular bone was stained in blue and fibrin-containing tissue in pink; (**I**,**J**) illustrate the results from our morphometric analysis of the regenerated tissue covering the calvarial defect; (**I**) documents the relative calvarial defect area by dividing the original area of the defect with a diameter of 5 mm by the area covered in the control, fibrin gel, fresh and lyophilized PRF treatment groups, and (**J**) represents the morphometric analysis of bone *versus* fiber ratio in all four groups. **: *p* < 0.01, and ***: *p* < 0.001.

To investigate the application of lyophilized PRF in bone regeneration, critical size cranial bone defects were created and defects were covered with either fibrin gel, fresh or lyophilized PRF. Six weeks after surgery, three-dimensional CT scans of the cranium revealed an apparently open defect in the fibrin gel group, while the defect area was reduced in fresh PRF group and the defect nearly closed in the lyophilized PRF group (Figure 6A,C,E,G). Application of lyophilized PRF resulted in 97% bone defect coverage, compared to 84% when using fresh PRF, 64% when using fibrin alone, and 16% when the defect was left without scaffold after six weeks (Figure 6A,C,E,G,I). Image analysis demonstrated 62.13% ± 1.89% area coverage of mineralized tissue after LPRF treatment, *versus* 43.91 ± 1.35 after fresh PRF treatment, 31.65 ± 5.84 after fibrin treatment, and 16.74 ± 8.65 without application of any scaffold (Figure 6B,D,F,H,J). Differences in the bone *versus* fiber area ratio were highly significant (*p* < 0.001) for LPRF and PRF versus control and highly significant (*p* < 0.01) for fibrin versus untreated control (Figure 6J). Moreover, lyophilized PRF increased the diameter of bone trabeculae compared to fresh PRF and fibrin by 25% (6.0 ± 2.1 μm fibrin, 6.1 ± 2.1 μm fresh PRF *versus* 8.6 ± 2.5 μm fresh PRF) (Figure 6D,F,H), and both lyophilized and fresh PRF resulted in long and interconnected bone trabeculae compared to short disrupted trabeculae after application of fibrin scaffold alone (Figure 6F,H *versus* Figure 6D).

## 3. Discussion

The purpose of the present study was to determine the suitability of lyophilized Platelet-rich fibrin (LPRF) as a tissue engineering scaffold for lost periodontal tissues and craniofacial bone. Therefore, a thorough comparison of cell proliferation, migration, mineralization behavior, and mineralization gene expression between fresh and lyophilized PRF *versus* DMEM medium was conducted in dental follicle cells, periodontal progenitors, and alveolar bone cells. We have also tested the effect of lyophilized PRF on cell homing and collagen synthesis in subcutaneous implants and determined its effect on cranial bone regeneration. Together, data from the present study indicate that lyophilized PRF surpasses fresh PRF as a scaffold material in terms of proliferation induction, osteogenic differentiation, and tissue integration.

Our studies indicated that both cell proliferation and cell migration rate of periodontal progenitors were increased significantly after the use of lyophilized PRF when compared to fresh PRF. We attribute the enhanced proliferation and migration in LPRF scaffold to two reasons, (i) the increased pore size in LPRF scaffolds and (ii) the improved release of growth factors and cytokines in LPRF scaffolds when immersed in cell culture media. The use of lyophilized platelets to provide hemorrhagic patients with a stable hemostatic agent to stop bleeding goes back more than 50 years ago [26,27]. Several years ago, the process of lyophilization was applied toward platelet rich fibrin scaffolds [24], but so far these scaffolds have not been tested in tissue engineering applications. Our recent successful application of PRF for periodontal regeneration and alveolar bone augmentation [25] prompted our interest in testing the applicability of a plasma preparation with enhanced shelf-life for periodontal tissue engineering and bone regeneration. As it turned out, LPRF featured 13.4-fold larger pore size, and this enhanced pore size is known to affect cell behavior, including adhesion, proliferation and gene expression [18,28–30]. Lyophilized PRF preparation not only enhances pore size and the ability of cells to migrate and proliferate within the scaffold, but also allows for slow release of growth factors via the surface of the scaffold preparation. Another benefit of lyophilized PRF was the degradation of scaffold fibers after seven days of implantation, while the scaffold consisting of fresh PRF showed little or no evidence of degradation. Evidence for the benefit of lyophilized platelet preparation of platelet growth factor release has been provided in previous studies [31,32], and a similar mechanism might contribute to the increase of LPRF on cell migration and proliferation when compare to fresh PRF.

Our data demonstrated that lyophilized PRF dramatically promoted mineralization induction exclusively in alveolar bone progenitors, but not in periodontal progenitors or dental follicle cells through an almost 5-fold increase in RunX2 expression and a corresponding reduction in MGP expression. The effect of fresh PRF on periodontal regeneration and alveolar bone augmentation had been characterized in a previous study from our group, which provided the baseline DMEM and fresh PRF data for proliferation, migration, mineralization, and gene expression levels presented here [33]. In the present study, we report that lyophilized PRF is 1.3-fold–1.4-fold more effective in stimulating RunX2 expression than fresh PRF, which has been already established as an effective means for bone regeneration, suggesting that LPRF is an even more efficient scaffold for bone tissue engineering. The effect of LPRF on RunX2 expression was unique to alveolar bone cells, and its effect on mineralization behavior was detectable both in alveolar bone cells and periodontal progenitors, while there was no effect on dental follicle cells, suggesting that LPRF promoted mineralization in a tissue-specific fashion. We propose that the tissue-specific effect of LPRF on osteoblasts may be due to the effect of fibrin on reparative osteogenesis [28,34], likely aided by osteogenesis-promoting growth factors.

These encouraging results related to the use of LPRF on cell proliferation, migration, and mineralization behavior encouraged us to conduct a series of *in vivo* studies to verify the efficacy of LPRF as a novel scaffold for tissue regeneration and osteoinduction. These studies indicated that LPRF was a superior scaffold when tested in subcutaneous implants allowing for extensive penetration and invasion by surrounding cells, while fresh PRF remained separated from the surrounding environment. We interpret the benefits of the freeze-drying process on improved tissue integration to be a result of the increased pore size in LPRF [18,29,35]. Moreover, when applied in cranial bone defects, the combination of tissue integration and bone inductive qualities proved beneficial, resulting in substantially increased bony defect coverage and a 2-fold increase in trabecular diameter in regenerated bony defects. These results suggest that LPRF preparations provide a promising avenue for the future treatment of craniofacial bone defects, which remains a challenging area for reconstructive surgery [36].

## 4. Experimental Section

### 4.1. Preparation of Fresh PRF, Lyophilized PRF, and Conditioned Medium

Fresh blood samples containing 10 mL blood in 10 mL coated glass tubes without anticoagulants were collected from the pig precaval vein. All pigs were female with a mean age of 3.1 months (range from 2.9 to 3.5 months). The mean whole blood platelet count of was 3.9 × 10^4^/μL (3.2 × 10^4^ to 4.66 × 10^4^/μL). All experiments in this study were approved by the Ethics Committee of the University of Illinois at Chicago, IL, USA. As described in previous studies, samples were immediately centrifuged at 2100 rpm (approximately 400 g) for 12 min using a Beckman tabletop centrifuge. The PRF clots were collected between the red corpuscles at the bottom of the centrifuge tube and platelet-poor acellular plasma at the top of the centrifuge tube, and then were gently compressed to form a membrane. For the preparation of lyophilized PRF, PRF membranes were frozen and stored at −80 °C. The frozen PRF was then freeze dried overnight using a Labconco lyophilizer at −51 °C (Free Zone, Labconco, Kansas City, MO, USA). To prepare conditioned medium, either fresh or lyophilized PRF membranes were soaked in 5 mL fresh DMEM medium without fetal bovine serum in 6-well cell culture plates. The conditioned medium was collected every 48 h and fresh medium was added into the wells after collection.

### 4.2. Isolation of Human Dental Mesenchymal Stem Cells

Mesenchymal stem cells were collected from healthy human teeth (patients ranging from 12 to 15 years) extracted for orthodontic reasons in accordance with a human subjects protocol approved by UIC’s Institutional Review Board and the Office for the Protection of Research Subjects. Dental follicle (DF), dental pulp (DP), alveolar bone (AB) and periodontal ligament (PDL) were dissected from the developing tooth organs, and mesenchymal stem cells were isolated from the dental tissues after digestion with collagenase/dispase as described before [37].

### 4.3. Tissue Processing

Athymic nude mice and nude rats were bred at the University of Illinois at Chicago or at Jilin University in strict accordance with the recommendations on the Guide for the Care and Use of Laboratory Animals of the National Institutes of Health. The protocols were approved by the Committee on the Ethics of Animal Experiments of both Universities. Implants or calvarial bones were dissected and fixed with 10% formalin at 4 °C.

### 4.4. Scanning Electron Microscopy

Fresh and lyophilized PRF membranes were fixed in 10% formalin solution immediately after preparation. After dehydration, samples were coated with gold palladium using a sputter coater, and examined using a scanning electron microscope (Hitachi S-3000N Scanning Electron Microscope, Tokyo, Japan).

### 4.5. Proliferation Assay

PDL, DF and AB were seeded into 96-well tissue culture plates at a concentration of 1000 cells per well. Plates were placed in the incubator for 8 h. After cell attachment, each well was washed twice with sterile phosphate buffer saline solution (PBS). Subsequently, lyophilized PRF and fresh PRF conditioned medium + 10% FBS, as well as DMEM + 10% FBS (control) were added to each well. At each time point the MTT assay (MTT, Sigma Chemical Co., St. Louis, MO, USA) was performed. The spectrophotometric absorbance (optical density) was read at 540 nm using a microplate reader (Power Wave 200 microplate scanning spectrophotometer, Bio-Tek Instruments, Winooski, VT, USA).

### 4.6. Chemotaxis Assay

Eight μm pore size fluoroblok inserts (Falcon/Becton Dickinson, St. Jose, CA, USA) were used to analyze the migration of different cells towards different types of medium. DF, PDL and AB cells were cultured in DMEM medium with 1% serum overnight and then seeded into inserts at concentration of 5 × 10^4^ cells/insert. The lyophilized PRF and fresh PRF conditioned medium, or DMEM were added into lower chambers separately. Plates were placed in the incubator for 12 h. After rinsing twice, inserts were stained with 1% DAPI (Invitrogen, Molecular Probes™, Grand Island, NY, USA) in dark. The numbers of migrated cells was counted using a fluorescence microscope.

### 4.7. Alkaline Phosphatase (ALP) Activity Assessment

PDL, DF and AB were seeded into 6-well cell culture plates at a concentration of 10^4^ cells/well and the plates were placed into a CO_2_ incubator for 8 h. After cell attachment, the fresh and lyophilized PRF conditioned media were added into different wells under osteogenic induction conditions (10^−8^ M dexamethasone, 10 mM β-glycerophosphate and 50 μg/mL ascorbic acid). After 7, 14, and 21 days of co-culture, cells were fixed and stained with NBT/BCIP (Roche Diagnosis GmbH, Mannheim, Germany) after rinsing with PBS. The color density of the stained area was measured using the Image-Pro Plus 6.0 software (Media Cybernetics, Rockville, MD, USA).

### 4.8. Quantification of Mineralization Nodules

DF, PDL and AB cells were seeded as described above in the ALP activity experiment. After 7, 14, and 21 days co-culture, cells were fixed and stained using Alizarin Red S (Sigma, Sigma Chemical Co., St. Louis, MO, USA). The color density of matrix mineralization was measured using the Image-Pro Plus 6.0 software.

### 4.9. Gene Expression Assay

DF, PDL and AB cells were seeded as described above in ALP activity experiment. After 7, 14, and 21 days co-culture, cells were harvested for RNA isolation. After transcription of cDNA, quantitative real-time PCR was performed using Cyber Green Master Mix and ViiA™ 7 Real-Time PCR System (Applied Biosystems, Grand Island, NY, USA). Primer sequences were as follows: Runx2: 5′-TTACTTACACCCCGCCAGTC-3′ (sense), 3′-CACTCTGGCTTTGGGAAGAG-5′ (antisense); Mgp: 5′-CCCTCAGCAGAGATGGAGAG-3′ (sense), 3′-GCTTCCCTATTGAGCTCGTG-5′ (antisense); β-Actin: 5′-GCATGGGTCAGAAGGATTCCT-3′ (sense), 3′-TCGTCCCAGTTGGTGACGAT-5′ (antisense), (Integrated DNA Technologies, Skokie, IL, USA).

### 4.10. Subcutaneous Implant

Fibrin gel, fresh and lyophilized PRF were cut into 5 mm squares and implanted into the subcutis of athymic nude mice (male, six weeks of age) under anesthesia with ketamine (100 mg/kg) and xylazine (5 mg/kg). Also this study was approved by the University of Illinois at Chicago Animal Care Committee. After 7 and 14 days of implantation, nude mice were sacrificed and implants were removed for histological sectioning and Hematoxylin and Eosin staining.

### 4.11. Bone Regeneration in the Rat Critical Size Calvarial Defects

Athymic nude rats (275 g body weight) were anesthetized by intraperiotoneal injections of ketamine (100 mg/kg)/xylazine (5 mg/kg) as approved by the Ethics Committee of the University of Illinois at Chicago, USA. After shaving the skin, the calvaria were sectioned longitudinally to expose the periosteum from the nasal bone in caudal direction to the mid-sagittal crest. The periosteum was displaced with surgical tools, and a 5 mm defect was created in the parietal bones with a hand-held trephine drill, exposing the encephalic dura mater [38]. The bone defect was filled with a 5 mm fibrin gel, fresh or lyophilized PRF. After 6 weeks of the surgery, the experiment was terminated and the cranium was dissected and fixed to proceed with micro-CT.

### 4.12. Micro-CT Analysis

To visualize regenerated tissues, rat calvarial bones were analyzed using microcomputed tomography (micro-CT). For this purpose, 3D X-ray CT images were acquired using an Xradia MicroXCT 400 (Xradia, Concord, CA, USA). Briefly, a 1024 × 1024 image matrix size over a 5.12 mm field of view was selected to create an isotropic voxel size of 5 microns. A total of 1024 slices were acquired for each mandible. No filtering processes were applied after the scan and reconstruction. During the scans, a 30 KeV, 6 Watt X-ray beam (Xradia, Concord, CA, USA) was generated to image the samples; a 5 s exposure time was used for each of the hundreds of projection images with a 0.25 degree step angle.

### 4.13. Statistical Analysis

Statistical analysis (ANOVA) was performed using the SPSS 13.0 software (version 10.0; Chicago, IL, USA). Data were expressed as mean ± SEM. For all tests, statistical significance was assigned when *p* < 0.05. Experiments were repeated three times to ensure reproducibility.

## 5. Conclusions

Together, our findings indicate that lyophilized PRF promotes Runx2-mediated osteogenic lineage commitment in alveolar bone cells, but not in dental follicle progenitors and only marginally in periodontal progenitors. Moreover, these studies support the application of lyophilized PRF as a biomimetic scaffold for craniofacial bone regeneration and mineralized tissue engineering. Compared to fresh PRF, lyophilized PRF has the benefit of improved storage capacity, increased osteogenic potential, and better tissue integration into the wound site.
